# On the Action of Lead in Distilled and River Water

**Published:** 1845-06

**Authors:** Richard Phillips


*On the Action of Lead in distilled and River Water.* By Richard Phillips, Junr.—The author considers, from the action which is the subject of his paper, the following points of interest to arise: 1st, whether it is necessary that the lead should be exposed to the air, or whether the action would take place if it were below the surface of the water; 2nd, whether pure or distilled water, deprived of all atmospheric air, has any action or lead; 3rd, as to what is the nature of the white precipitate; 4th, if, besides this white precipitate, any soluble salt of lead is found; and 5th, as to which of the salts contained in river-water the non-formation of the white precipitate is due.

He proceeds to notice the remarks of Dr. Christison and Colonel Yorke on these subjects, and then gives his own experiments.

As to the first. In bottles containing distilled water were placed two pieces of lead of nearly equal size. In both a white precipitate occurred, but in that in which the lead had been exposed above the surface of the water, a far greater quantity than in the one where the lead had been exposed above the surface of the water; and he concludes from this experiment, that although it is not necessary that the lead should be exposed above the surface of the water for the action to take place, still by offering a passage to the gases contained in the atmosphere it accelerates it.

The author observes, "I have before stated, Dr. Christison does not describe the method by which he performed his experiment on the non-solubility of lead in pure distilled water; and although I agree with him as to the fact, as I found some difficulty in ascertaining it, I will describe the process I followed. I found that on boiling pure distilled water so as to deprive it of all atmospheric air, that when it had regained its former temperature, and was placed in a well-stoppered bottle with a piece of lead, the bottle being perfectly full, although no action was apparent, and the lead remained almost as bright as when first placed in the bottle, yet if a little hydrosulphuric acid was added to the water, it instinctly became of a brown color, showing the presence of lead, the water having on cooling absorbed sufficient atmospheric air to act on the lead. If, on the contrary, the water was placed in the bottle when warm, and the lead immediately added, and the bottle stoppered, on cooling a vacuum was formed, which it was impossible to maintain, and the lead was acted upon. I therefore repeated the last experiment, but took the precaution, when the bottle was quite full and no vacuum had formed, to plunge the stopper under mercury, and to pour water above the mercury. The experiment was allowed to remain for some months; the lead retained its original brightness; and on breaking off the neck of the bottle, it being impossible on account of the vacuum to take out the stopper, not the slightest effect was produced on the liquid by hydrosulphuric acid, thus proving that pure water, without the presence of air, has no action on lead.

"From my experiments I am led to the same conclusion as Colonel Yorke, as to the manner of the formation of the white precipitate; in fact, I have obtained small masses of crystalline scales, which have formed on the surface of lead, which have dissolved without the slightest effervescence in acetic acid. I do not think, however, any fixed composition can be assigned to this substance, as on repeating his experiment of drying it under the air-pump upon two different specimens, one of which had been kept some years, and the other only a few weeks, in the former a much larger amount of carbonic acid was found than in the latter, showing that the precipitate had no definite composition, but that it depended, as to the quantity of carbonic acid, on the length of time it had been exposed in the solution.

"As to the existence of either of the salts of lead mentioned in a soluble state, I must confess that I differ with the two authorities I have named, and believe that what they consider to have dissolved is merely the hydrated oxide of lead held in a state of mechanical suspension, and for the following reasons:—I found in repeating an experiment of Colonel Yorke's, that of precipitating hydrated oxide of lead and washing it with warm distilled water, that the filtered liquor gave a brown precipitate with hydrosulphuric acid, after all the soluble salts formed had been washed away; but that on refiltering a portion of it through more than one filter, not the slightest discoloration was given to the liquor by the test; and as Colonel Yorke considered that the soluble salt was the hydrated oxide, a greater quantity of which would exist, as already shown, at the commencement of the action of the lead on the water, I placed a piece of lead in distilled water, and carefully tested the filtered liquor daily, for some weeks, with hydrosulphuric acid, but in no case could I find the slightest discoloration given to it. Now as the test is so delicate as to be capable of discovering, according to Pfaff, a 100,000th part of the metal in solution, if, as Colonel Yorke states, a 10,000th part of the oxide is held in solution, the test would of course have distinctly shown it.

"Dr. Christison considering that the action of the formation of a soluble salt of lead was due to the carbonate of lead becoming converted into bicarbonate by long exposure, I tested the liquid from one of the experiments I have before mentioned, when the lead in distilled water had been exposed to the action of the atmosphere for six years, and could not detect the slightest discoloration after the solution had been carefully filtered. I therefore conclude from these experiments, and I believe Dr. Thomson has previously arrived at the same result, that no soluble salt of lead is formed by the action of atmospheric air on lead in distilled water."

As to the last question, "to which of the salts contained in river-water the non-formation of the white precipitate is due," the author states that it is the sulphate of lime, and considers that the chloride of sodium, which has been generally supposed to be capable of exercising this preservative power, does not possess it, on account of its forming when decomposed a salt of lead partly soluble.

In conclusion, he remarks that the non-action of rain-water which had fallen, after a long continuance of dry weather, on lead, is probably due to the small amount of carbonate of ammonia which is contained in atmospheric air.—*London Chemical Gazette.*

